# Dysbiosis of the microbiota in neurocritically ill patients associated with coma and death: ammonia as a potential missing link

**DOI:** 10.1186/s13054-019-2688-y

**Published:** 2019-12-11

**Authors:** Patrick M. Honore, Aude Mugisha, Leonel Barreto Gutierrez, Sebastien Redant, Keitiane Kaefer, Andrea Gallerani, David De Bels

**Affiliations:** ICU Department, Centre Hospitalier Universitaire Brugmann, Place Van Gehuchtenplein 4, 1020 Brussels, Belgium

Xu et al. conclude that changes in gut microbiota in neurocritically ill patients seem to have an impact on their mortality [1]. We would like to add some comments. The authors described an overgrowth of opportunistic pathogens defined as dysbiosis in patients with neurocritical illness. This study had similar results to other studies regarding the appearance of pathogens and disappearance of commensals [2, 3]. Further, the authors said that dysbiosis of the microbiota in neurocritical patients can be reasonably presumed to increase the risk of infection, undernutrition, and unconsciousness [1]. Here we would like to link dysbiosis and unconsciousness, where increased production of ammonia may play an important role. Indeed, bacteria residing in the human gut produce urease which is beneficial in healthy hosts but pathogenic in hosts with liver disease [4]. Urea produced by the liver is both excreted in urine and transported into the colon, where it is hydrolyzed by bacterial urease into carbon dioxide and ammonia [4]. Circulating ammonia is correlated with brain damage in patients with acute or chronic liver disease resulting in hepatic encephalopathy. In Xu’s study, nearly 40% of the patients had liver disease [1]. It is somewhat unfortunate that blood ammonia was not measured. This would have been of great utility to better interpret the results of their study [1, 4].

Xu also suggested that critical illness could lead to microbial translocation, potentially explaining the association between specific pathogens and mortality [1]. Another valid explanation, knowing that there was an overgrowth of enterobacteriaceae in this study [1], could be the translocation of endotoxin [5]. Indeed, translocation of endotoxin can trigger sepsis, septic shock, and secondary peritonitis [5]. This may have been an important contributing factor in this study, particularly in the 40% of patients who had liver disease [1] and therefore were less capable of filtering endotoxin [1, 5]. Measurement of endotoxin levels could be a useful addition in further studies.

## Data Availability

Not applicable.

## Abbreviation

ICU: Intensive care unit
